# Rates of Diagnoses Indicating Opioid Dependence After Hospice Live Discharge: A National Study

**DOI:** 10.1093/geroni/igaa057.057

**Published:** 2020-12-16

**Authors:** Thomas Christian, Ian Breunig, Joan Teno, Pedro Gozalo, Michael Plotzke

**Affiliations:** 1 Abt Associates, Cambridge, Massachusetts, United States; 2 Oregon Health & Science University, Portland, Oregon, United States; 3 Brown University, Providence, Rhode Island, United States

## Abstract

Opioids are an important tool for managing Medicare Hospice beneficiaries’ pain and symptoms. Little is known about the prevalence of opioid dependence among patients discharged alive from hospice. Using 100% Medicare hospice, acute inpatient, and Part B claims from Federal Fiscal Years (FY) 2017-2018, we identified hospice beneficiaries that were discharged alive during FY2017-2018 and associated with diagnosis codes in subsequent health care incidents indicating opioid dependence. We used a crosswalk from the Agency for Healthcare Research and Quality to determine which codes represented opioid dependence. We characterized beneficiaries and their hospice providers using information from the Medicare Enrollment Database and Provider of Services file. There were 468,204 live hospice discharges during FY2017-2018, among which 9,282 (2.0%) were associated with subsequent health care events including a diagnosis code signifying opioid dependence. Post-hospice opioid diagnoses were more frequent among beneficiaries who were younger (for ages <65 relative to 85-89; Adjusted Odds Ratio [AOR]=6.23, 95% Confidence Intervals [CI] 5.73-6.77); dual-eligible (AOR=1.40, 95% CI 1.34-1.47); and relative to cancer, diagnosed with lung (AOR=1.83, 95% CI 1.72-1.95), heart (AOR=1.23, 95% CI 1.14-1.34), or liver diseases (AOR=1.25, 95% CI 1.10-1.42). Beneficiaries with opioid incidents tended to have survived much longer after hospice (150.9 days with opioid incidents vs. 13.6 days no opioid-related incidents). The states with the highest rates of post-hospice opioid-incidents per 10,000 live discharges were Kentucky (435.2), Wyoming (386.0), Tennessee (359.3), Washington (345.6), and Idaho (342.9). Further monitoring can ensure that hospice beneficiaries receive appropriate care during and after hospice enrollment.

